# Effects of physical exercise on skeletal muscles of rats with cerebral ischemia

**DOI:** 10.1590/1414-431X20198576

**Published:** 2019-11-28

**Authors:** R.T.R. Melo, L.C.M. Damázio, M.C. Lima, V.G. Pereira, B.S. Okano, B.S. Monteiro, A.J. Natali, R.J. Del Carlo, I.R.S.C. Maldonado

**Affiliations:** 1 Departamento de Biologia Celular e Estrutural, Universidade Federal de Viçosa, Viçosa, MG, Brasil; 2 Departamento de Medicina, Universidade Federal de São João del-Rei, São João del-Rei, MG, Brasil; 3 Departamento de Morfologia, Universidade Federal de São João del-Rei, Divinópolis, MG, Brasil; 4 Departamento de Medicina Veterinária, Universidade Federal de Viçosa, Viçosa, MG, Brasil; 5 Departamento de Educação Física, Universidade Federal de Viçosa, Viçosa, MG, Brasil

**Keywords:** Muscles, Cerebral ischemia, Exercise, Movement, Rats, Atrophy

## Abstract

Physical exercise is a known preventive and therapeutic alternative for several cerebrovascular diseases. Therefore, the objective of the present study was to evaluate the motor performance and histomorphometry of the biceps brachii, soleus, and tibialis anterior muscles of rats submitted to a treadmill training program prior to the induction of cerebral ischemia via occlusion of the middle cerebral artery (OMCA). A total of 24 Wistar rats were distributed into four groups: Sham-Sed: sedentary control animals (n=6), who underwent sham surgery (in which OMCA did not occur); Sham+Ex: control animals exercised before the sham surgery (n=6); I-Sed: sedentary animals with cerebral ischemia (n=6); and I+Ex: animals exercised before the induction of ischemia (n=6). The physical exercise consisted of treadmill training for five weeks, 30 min/day (5 days/week), at a speed of 14 m/min. The results showed that the type-I fibers presented greater fiber area in the exercised ischemic group (I+Ex: 2347.96±202.77 µm^2^) compared to the other groups (Sham-Sed: 1676.46±132.21 µm^2^; Sham+Ex: 1647.63±191.09 µm^2^; I+Ex: 1566.93±185.09 µm^2^; P=0.0002). Our findings suggested that the angiogenesis process may have influenced muscle recovery and reduced muscle atrophy of type-I fibers in the animals that exercised before cerebral ischemia.

## Introduction

Cerebrovascular accidents, also known as strokes, are neurological disorders that belong to the group of cerebrovascular diseases that are currently the primary cause of disability in adults and the second leading cause of death worldwide (1–3). Depending on the triggering factor, strokes are classified as ischemic or hemorrhagic. Ischemic strokes correspond to more than 80% of cerebrovascular accidents and occur by obstruction of the main arteries that carry blood to the brain. Considering that the brain is an organ with a high metabolic rate that depends on the continuous supply of oxygen and glucose via blood, the severe or complete reduction of blood flow (ischemia) results in the development of instantaneous functional and biochemical deficiencies in neurons (4).

In the brain, the nervous tissue undergoing ischemia endures a series of complex and intricate events, which together are known as the ‘ischemic cascade'. Minutes after vascular occlusion, neuronal depolarization occurs, leading to excessive release, which is followed by failure to reuptake the neurotransmitter glutamate, increases in intracellular calcium levels, excessive production of reactive oxygen species, depletion of antioxidant enzyme levels, and the production of arachidonic acid and inflammatory mediators, as well as activation of second messengers involved in neuronal death signaling. Neurons that sustain injury in the center of the infarction area do not regenerate, thus producing neurological deficits that are often severe and permanent (5).

Clinical signs following an ischemic stroke may include deficiencies of the motor and sensory systems, cognitive and perceptual difficulties, and emotional alterations. Among the resulting forms of motor damage are: muscle weakness; muscular atrophy; hemiparesis, which consists of muscle paralysis on the opposite side of the body to the area of the brain lesion; lack of motor coordination, and spastic hypertonia of the upper and lower limbs on the opposite side of the lesion (6). These motor system impairments are due to nerve fiber degeneration in the corticospinal tract (6) as a result of injuries that may have occurred in the cerebral cortex, internal capsule, and brainstem.

Muscular atrophy is characterized by a decrease in muscle mass, cross-sectional fiber area, and myofibrillar protein content (7). Animal studies have provided evidence of muscle weight reduction on both the affected side (contralateral to the brain lesion) and the unaffected side during the 7 days following ischemic stroke (8,9). Hafer-Macko et al. (10) demonstrated that the expression of tumor necrosis factor α (TNF-α) is enhanced in the skeletal muscles of ischemic stroke individuals. Such an increment contributes to muscular atrophy by inhibiting protein synthesis and transcriptional regulation of myofibrillar gene expression by increasing protein degradation through ubiquitinated proteinases and by cell death via apoptosis.

Hemiparetic individuals have benefited from muscle strengthening programs, enhancing their performance (11). Engardt et al. (12) reported that physical exercise is associated with reduced muscular atrophy and significant improvements in motor function, including knee extension and flexion movements, of all the affected musculature. Several studies have identified advantages of physical exercise practiced before cerebral ischemia, denoting a neuroprotective effect on the brain, as well as benefits in motor performance (13–15).

The present study aimed at evaluating the effects of physical exercise in rats prior to the induction of cerebral ischemia regarding the motor performance and structure of the following skeletal muscles: biceps brachii, type-II muscle fibers with flexor activity in the thoracic limbs, and tibialis anterior and soleus, pelvic limb muscles that are type-II and type-I muscle fibers, respectively. The tibialis anterior and soleus muscles represent a particularly important agonist/antagonist pair in the “fallen foot” motor deficit (16), often observed in humans following ischemic stroke.

Type-I fibers (also regarded as ‘red fibers') present lower mATPase activity, slower ATP degradation rates, and are used in exercises that require slow contraction. These fibers have higher concentrations of oxygen and myoglobin and a significant number of mitochondria and enzymes responsible for the oxidation of fatty acids. Type-II fibers (also known as ‘white fibers') show higher activity of the mATPase enzyme than type-I fibers and a more substantial rate of ATP degradation. They are less resistant to fatigue and present inferior concentrations of myoglobin and mitochondria but contain numerous myoneural plaques (17).

Despite the reasonable number of experimental studies on cerebral ischemia available in the literature, assessments relating to exercise, cerebral ischemia, and histology of skeletal muscle tissue remain scarce.

## Material and Methods

This study was approved by the Ethics Committee on Animal Experimentation of the Federal University of Viçosa, Brazil (32/2011). The procedures were conducted in accordance with the International Guiding Principles for Biomedical Research Involving Animals and the Brazilian College of Animal Experimentation.

### Animals

A total of 24 male Wistar rats (*Rattus norvegicus*), with 30 days of age and an average weight of 140 g, were obtained from the Bioterium of the Center of Biological Sciences and Health of the Federal University of Viçosa, CCB/UFV. The animals were housed in collective cages (five animals per cage) at the BioEx (Laboratory of Exercise Biology) of the Department of Physical Education, CCB/UFV, under controlled conditions of temperature and photoperiod (25°C and 12-h light/dark cycle), and provided with water and feed *ad libitum*.

**Figure 1:**
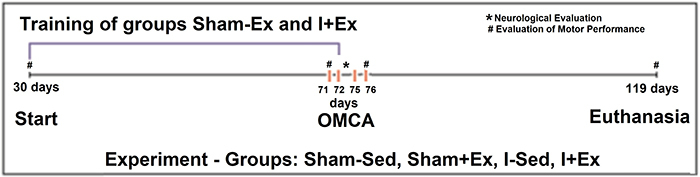
Design of experiment I, highlighting the main procedures conducted. At 30, 71, 76, and 119 days, motor performance was evaluated. At 72 days, occlusion of the middle cerebral artery (OMCA) was performed. The neurological evaluation was performed during the period of three days - 72 to 75 days. Sham-Sed: Sedentary control group; Sham+Ex: Control group exercised before the sham surgery; I-Sed: Sedentary ischemic group; I+Ex: Ischemic group exercised before OMCA surgery.

The training period lasted five weeks, and surgery for the induction of ischemia was performed when the animals were 72 days old. The following groups were established for the experiment: Sham-Sed group (n=6): control animals that underwent sham surgery (without induction of cerebral ischemia) and that did not exercise (sedentary); Sham+Ex group (n=6): control animals exercised on a treadmill before sham surgery; I-Sed group (n=6): sedentary animals with cerebral ischemia; I+Ex group (n=6): animals exercised on a treadmill prior to the induction of cerebral ischemia (Figure 1).

The animals were euthanized 41 days after the induction of cerebral ischemia (day 119), and the animals were weighed at the beginning of the experiment and before euthanasia using a precision digital scale (SPLabor, Brazil) with two decimal places.

### Physical training

The treadmill training program (Insight Instrumentos, Brazil) was carried out for six weeks, the first of which was designated for animal adaptation, which consisted of the following: Day 1: treadmill speed of 10 m/min for 10 min with a slope of 0 degrees; Day 2: treadmill speed of 10 m/min for 10 min and a 3-degree slope; Day 3: speed of 11 m/min for 11 min and a slope of 3 degrees; Day 4: speed of 11 m/min for 11 min on a 5-degree slope mat; Day 5: speed of 12 m/min during 12 min on a 5-degree slope mat.

After the adaptation week, the animals were submitted to training for five weeks, at a speed of 14 m/min, five days per week, for 30 min/day (18), which corresponded to moderate intensity. The maximum stress test was performed on the animals and an average velocity of 14 m/min was observed.

Meanwhile, the sedentary animals exercised on the treadmill once a week for 5 min, at a speed of 5 m/min.

### Occlusion of the middle cerebral artery (OMCA)

Cerebral ischemia was induced via OMCA, according to Longa et al. (19). The animals were anesthetized using 4% isoflurane (Isoflurane^®^; Abbott Laboratory, USA) by inhalation, and isoflurane at a concentration of 2.5% and oxygen (1.5%) was given for anesthetic maintenance. A dose of atropine (5.0 mcg/100 g animal weight) was administered intraperitoneally to prevent cardiac arrhythmias and bronchial hypersecretion secondary to mechanical stimulation of the vagus nerve during the surgical procedure. For prophylactic antibiotic coverage, a veterinary pentabiotic (Zoetis, Brazil) (benzathine benzylpenicillin + benzylpenicillin procaine + benzylpenicillin potassium + dihydrostreptomycin+streptomycin) was administered intramuscularly (0.1 mL/rat).

After trichotomy of the ventral cervical region and subsequent asepsis with iodinated alcohol, a median sagittal incision was performed, and the tissues were moved away until visualization of the left common carotid artery (CCA) bifurcation. Next, the branch of the internal carotid artery (ICA) and the CCA were clamped. The external carotid artery (ECA) was ligated with cotton thread at its distal portion and sectioned with the aid of microphthalmic scissors. The occlusion wire was introduced into the left ECA and directed into the ICA. In the animals submitted to the sham surgery (controls), the occlusion wire was removed from the ICA, and the blood flow was released to the encephalon. In turn, in the ischemic animals, the wire continued to be inserted into the ICA. Two criteria were employed to determine if the occlusion wire was at the origin of the MCA: wire insertion at a distance of 20–22 mm from the CCA bifurcation and/or slight resistance concerning wire passage at that distance. The occlusion wire was maintained in place for 60 min, during which any adverse effects, including cardiac and respiratory arrest, bronchial hypersecretion or hypothermia, were monitored.

After removal of the occlusion wire, the ECA portion proximal to the CCA bifurcation was ligated using cotton thread, and the surgical incision was sutured with 3–0 Nylon. During the surgical procedure, body temperature was maintained between 36 and 38°C, with the animal on a thermal mattress.

During the first two hours after surgery, the animals were kept under a warm light to maintain normothermia. On the first postoperative day only, the bottom of the cage was covered with absorbent paper. Up to the fourth postoperative day, each rat received intraperitoneal doses of 3.0 mL of saline for ionic replacement.

### Neurological evaluation

Between 24 and 48 h after the induction of cerebral ischemia, the animals were classified regarding motor performance according to a severity index from zero to four (20). Zero denoted absence of apparent deficit; 1 for right anterior paw flexion; 2 for decreased grip of the right paw when suspended by the tail; 3 for spontaneous movement in all directions, but circular movement to the left when suspended by the tail; and 4 for spontaneous circular movement to the left. Ischemic animals presenting indexes three and four were included in the experiment (20).

### Evaluation of motor performance

In order to evaluate the motor performance of the animals, the following tests were adopted: false step and parallel bars. The first assay (false step test) was used to measure the motor coordination of the front paws, in which the animals were placed on a 100×50 cm grid with 3×3 cm subdivisions, suspended at 50 cm in height. Correct paw placement was analyzed for 3 min. Errors were considered every time the animal failed to touch the grid, and the right front paw passed through the empty space (9 cm^2^) (21).

The parallel bars test, in turn, was applied to measure hind leg motor coordination. The test artifact consists of two wooden platforms (30×20 cm and 40 cm high) joined by two parallel metal bars, measuring 115 cm in length and with a distance of 2.5 cm between each bar. The assessment was carried out for 5 min, and aversive stimulus (shock) was applied to the platform forcing the animal to move. Errors were considered when both legs were placed on the same bar and when they passed between the two bars or outside the bars (21).

The two tests were applied at the beginning of the experiment (30 days) to confirm whether the animals exhibited motor impairment. The rats were then evaluated prior to surgery, at 71 days, and 4 days after the procedures, at 76 days, before euthanasia.

### Microscopy

The animals underwent intramuscular injections of 0.2 mL of xylazine hydrochloride and 0.3 mL of ketamine hydrochloride. They were then transferred to a closed chamber containing isoflurane-soaked cotton. After this process, the individuals were placed in ventral decubitus for dissection and collection of the biceps brachii, soleus, and tibialis anterior muscles of the right limbs (paretic side in the ischemic animals). Each tissue was sectioned into several fragments to obtain samples that would be fixed in 4% paraformaldehyde solution in 0.1 M phosphate buffer at pH 7.2–7.4, and samples were rapidly frozen in N-hexane at –70°C, previously cooled in liquid nitrogen. Treatment with N-hexane promotes rapid freezing of the material, preventing the formation of ice crystals in the tissue. The paraformaldehyde-fixed fragments were submitted to inclusion processing in hydroxyethyl methacrylate (Historesin^®^, USA), while those frozen in N-hexane were stored in an ultra-freezer at –80°C to further obtain cryostat slices that would be submitted to succinate-dehydrogenase (SDH) histochemistry.

After fixation for 24 h in 4% paraformaldehyde in 0.1 M phosphate buffer, pH 7.2–7.4, the muscle fragments were stored in 70% ethyl alcohol solution. Later, they underwent dehydration in solutions containing increasing concentrations of ethanol (70, 80, 90, 95, and 100°GL) for impregnation and inclusion in hydroxyethyl methacrylate (Historesin^®^, USA).

Microtomy was performed using the model RM2255 Leica^®^ (Germany) rotating microtome, obtaining 4.0-μm-thick semi-serial cross-sections, with a minimum interval between cuts of 40 μm. The sections were stained with toluidine blue + sodium borate solution.

### SDH histochemistry

From each muscle fragment previously frozen in N-hexane, 12-μm-thick serial cross-sections were obtained using a model CM1850 LEICA^®^ cryostat, at -20°C. The sections were placed on histological slides for the SDH enzyme reaction. The SDH enzyme is a mitochondrial flavoprotein that catalyzes the oxidation of succinic acid into fumaric acid, and the histochemical demonstration of its activity is performed in the presence of succinate (substrate) and a redox indicator (tetrazolium). During the reaction, tetrazolium is converted into formazan (insoluble compound of navy-blue color, which precipitates inside the mitochondria). Type-I fibers have more mitochondria and, therefore, are highly positive regarding the SDH reaction compared to type-II fibers.

During the SDH reaction, the slides containing the sections were kept in a humid environment, in a Petri dish containing a filter paper disk moistened with distilled water. The sections were covered with incubation medium consisting of 0.2 M phosphate buffer + sodium succinate + nitro blue tetrazolium (NBT) at 37°C for 60 min. Afterward, they were treated with acetone solutions at 90, 60, and 30%, and the preparations were assembled with hydrophilic medium (Apathy’s syrup).

### Morphometry

Following the preliminary microscopy of the histological and histochemical (SDH) preparations, the images were captured using the QC CAPTURE program (version 3.1 for Windows) and the Olympus QColor-3^®^ digital camera system (Japan), coupled with a BX-60 Olympus^®^ microscope with 200× magnification.

In the biceps brachii and tibialis anterior muscle sections that were submitted to the SDH histochemical reaction, three microscopic images of the superficial region (rich in type-II fibers) and the deep region (rich in type-I fibers) of each muscle/animal were obtained. Regarding the soleus muscle, rich in type-I fibers and without regionalization of fibers I and II, three non-coincident images/animal were acquired. In turn, in the sections stained with toluidine blue + sodium borate, five non-coincident images were generated per muscle/animal.

The histomorphometric analysis of the obtained images was conducted with the aid of an image analysis program (Image Pro Plus, version 4.5 for Windows 98) and consisted of determining the frequency and area of type I and II muscle fibers in the sections submitted to the SDH reaction. The rate of the fibers is reported as a percentage. In order to determine fiber area, 50 cells were randomly and cross-sectionally measured by type (I, II)/muscle/animal, and the area values are reported in micrometers square (µm^2^).

In the sections stained with toluidine blue + sodium borate, histomorphometry involved the quantification of the following constituents: intact muscle fibers; muscle fibers with cytological changes (angulated fibers, nuclei displacement towards the center, fibers with cytoplasmic vacuolization); muscle fiber nuclei in a subsarcolemmal position and displaced to the center; muscular interstitium, and other elements (blood vessels, mast cells, and leukocytes). To this end, a test system with 225 points (grid with 225 intersections) was applied to each of the five captured images, totaling 1,125 points/muscle/animal. Since the analyzed image area represents 94,000 µm^2^, the total area analyzed per muscle per animal corresponded to 470,000 µm^2^. The values found in this histomorphometric analysis are reported as a percentage.

### Statistical analysis

The data were evaluated by two-way analysis of variance (ANOVA), followed by the Newman-Keuls *post-*test, at 5% significance, using the STATISTICA software (Dell, USA), for Windows 98. The obtained values are reported as means±SD.

## Results

### Body weight of the animals

No significant difference was observed among the groups regarding body weight at the beginning of the experiment (P=0.81). At the end of the assessment, however, the sedentary ischemic animals presented lower weight compared to the sedentary controls (P=0.45). Among the other groups, differences in final weight were not observed (P=0.72).

### Motor performance

All the animals in this study presented index 3 or 4 in the neurological evaluation. During the adaptation period, six animals refused to run and were therefore withdrawn from the survey.

**Figure 2:**
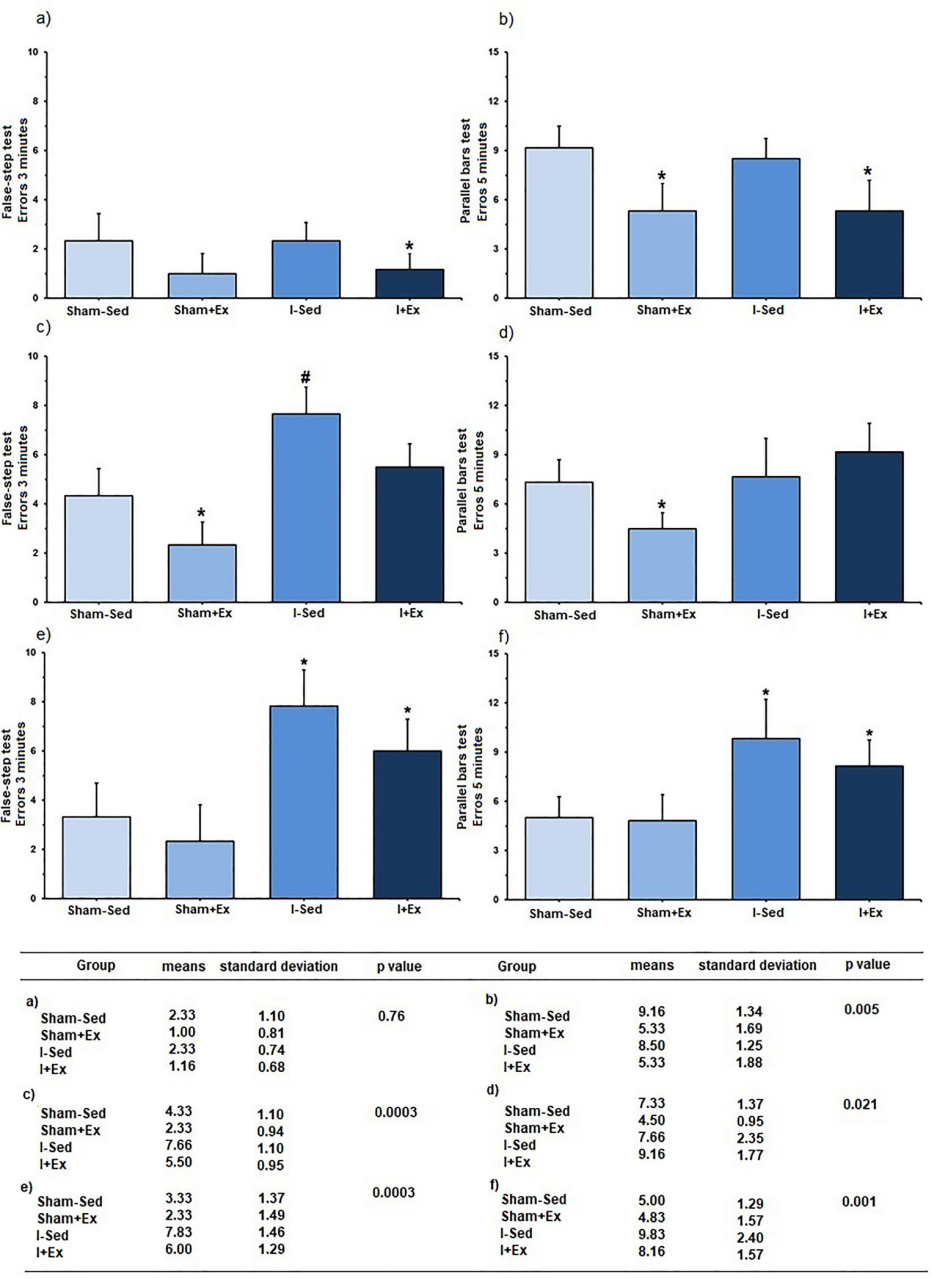
Evaluation of motor performance and table with numerical data. Errors (mean values) committed by the animals before (**a**,**b**) and after (**c**,**d**) the surgical procedures, and prior to euthanasia (**e**,**f**). **a**, *P=0.043 *vs* Sham-Sed group; **b**, *P=0.005 *vs* Sham-Sed and I-Sed groups; **c**, *P=0.0003 *vs* remaining groups. ^#^P=0.0002 *vs* Sham-Sed and I+Ex groups; **d**, *P=0.021 *vs* remaining groups; **e**, *P=0.0002 *vs* control groups; **f**, *P=0.001 *vs* control groups (ANOVA, followed by Newman-Keuls *post-*test). Sham-Sed: Sedentary control group; Sham+Ex: Control group exercised before the sham surgery; I-Sed: Sedentary ischemic group; I+Ex: Ischemic group exercised before occlusion of the middle cerebral artery surgery.

Prior to the surgical procedures, the number of errors committed by the animals of the I+Ex group was lower compared to the Sham-Sed group in the false step test (Figure 2a). Considering the other groups, however, no significant difference was observed (P=0.76). In the parallel bars test (Figure 2b), the animals that underwent training presented fewer errors compared to the sedentary groups (P=0.005). The mean values of the errors committed in the false step and parallel bars tests after surgery are shown in Figures 2c and 2d.

In the false step test (Figure 2c), a lower number of errors was committed by the exercised control animals compared to the other groups (P=0.0003). Also, a higher number of errors was observed in the sedentary ischemic group (I-Sed) compared to the sedentary controls (Sham-Sed) and the exercised ischemic group (I+Ex) (P=0.0003).

In the parallel bars test (Figure 2d), the exercised controls presented a lower number of errors compared with the other groups (P=0.021). A superior performance was expected in the exercised ischemic animals (I+Ex).

The mean values of the errors committed by the animals in the false step and parallel bars tests before euthanasia are shown in Figure 2e and f. In the false step test (Figure 2e), more errors were committed by the ischemic animals compared to the controls (P=0.0002). Similarly, in the parallel bars test (Figure 2f), the ischemic animals displayed an inferior yield (P=0.001).

### Frequency of type-I and type-II fibers

A higher frequency of type-I fibers was found in the superficial region of the biceps brachii of the exercised control group compared to the other groups (P=0.02). Considering the deep region of the biceps brachii, a higher frequency of type-I fibers was observed in the sedentary ischemic animals in relation to the other groups (P=0.007). Additionally, a lower frequency of type-I fibers (with a consequent increase in type-II fibers) was verified in the I+Ex group compared to the controls (Sham-Sed and Sham+Ex) (P=0.007).

In the soleus muscle, a higher rate of type-I fibers was observed in the animals that underwent physical training, as well as in those submitted to MCA occlusion. The ischemic groups presented a higher proportion of type-I fibers compared to the controls (P=0.0002), as did the trained animals compared to the sedentary groups (P=0.0002).

The distribution values of type-I fibers were observed in the superficial and deep regions of the tibialis anterior muscle, respectively, as follows: (P=0.095). Thus, a significant difference was not observed regarding the muscle fiber pattern.

### Areas of muscle fibers

Considering the type-I fibers, a larger fiber area was observed in the exercised ischemic group compared to the other groups (P=0.0002). Regarding type-II fibers, a larger fiber area was verified in the ischemic animals than in the control groups (P=0.002).

In the deep region of the biceps brachii, type-I fiber atrophy was noted in the sedentary ischemic animals in relation to the control groups (P=0.002). The exercised ischemic animals presented a considerably higher type-I fiber area than the sedentary ischemic group (P=0.0002). Regarding type-II fibers, a smaller fiber area was observed in the Sham+Ex group compared to the other groups (P=0.028).

A smaller area with type-I fibers was observed in the I-Sed and I+Ex groups compared to the controls (P=0.0002). The same result was found regarding type-II fibers, in which a smaller area was observed in the I-Sed and I+Ex groups in relation to the controls (P=0.004).

In the superficial region of the tibialis anterior muscle, changes were detected only with respect to the area of type-I fibers, where lower values were observed in the sedentary ischemic group compared to the control animals (P=0.023). In addition, a larger area of type-I fibers was verified in the exercised ischemic group compared with the sedentary ischemic group (P=0.006).

Considering the deep region of the tibialis anterior muscle, the Sham+Ex, I-Sed, and I+Ex groups presented lower values of type-I fiber area compared to the Sham-Sed group (P=0.001). Similarly, smaller areas were observed of type-II fibers in the Sham+Ex, I-Sed, and I+Ex groups compared with the sedentary control (P=0.008).

**Figure 3:**
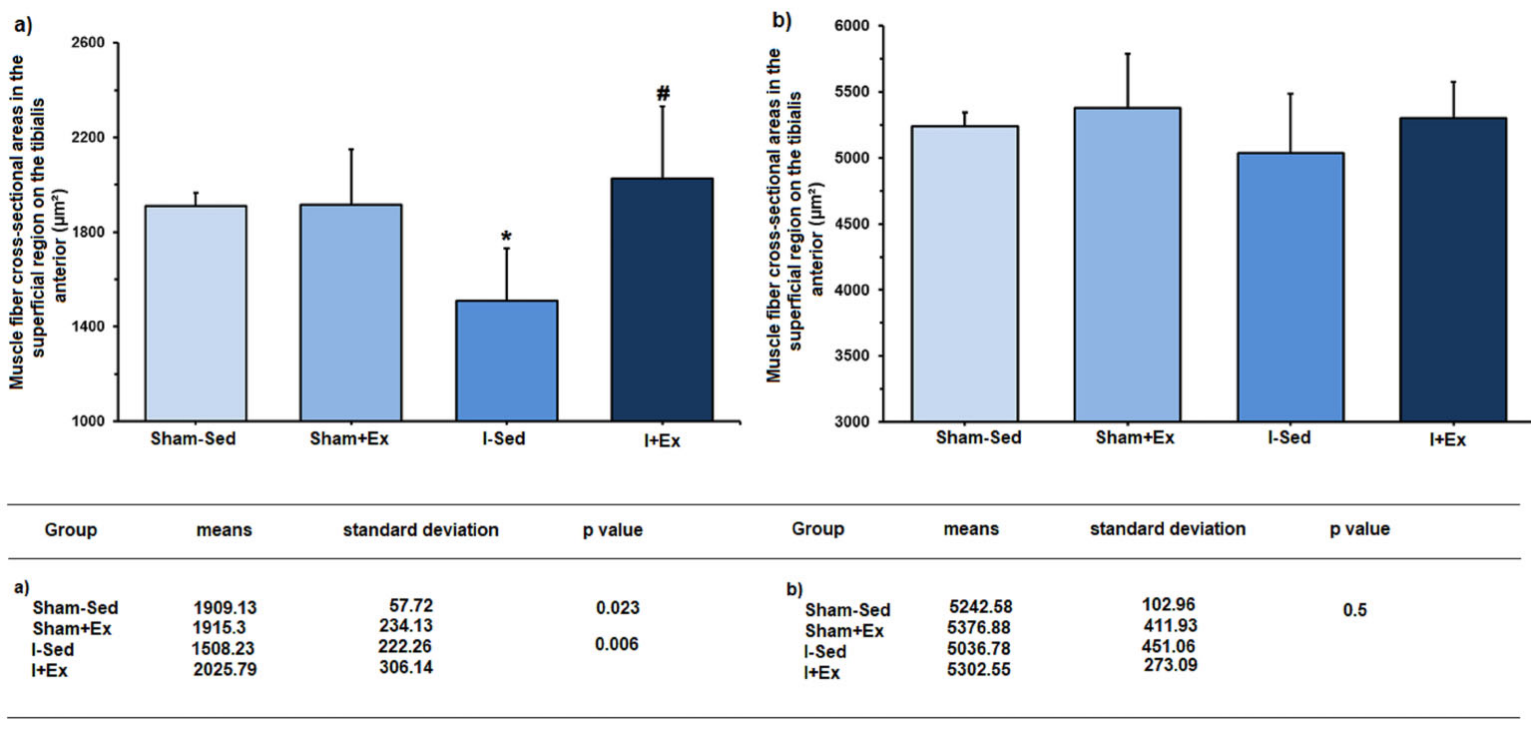
Muscle fiber cross-sectional areas in the superficial region of the tibialis anterior muscle and table showing the numerical data. **a**, Type-I fibers: *P=0.023 *vs S*ham-Sed group; ^#^P=0.006 *vs* I-Sed. **b**, Type-II fibers: P=0.5 Sham-Sed *vs* I-Sed group (ANOVA, followed by the Newman-Keuls *post-*test). Sham-Sed: Sedentary control group; Sham+Ex: Control group exercised before the sham surgery; I-Sed: Sedentary ischemic group; I+Ex: Ischemic group exercised before occlusion of the middle cerebral artery surgery.

**Figure 4:**
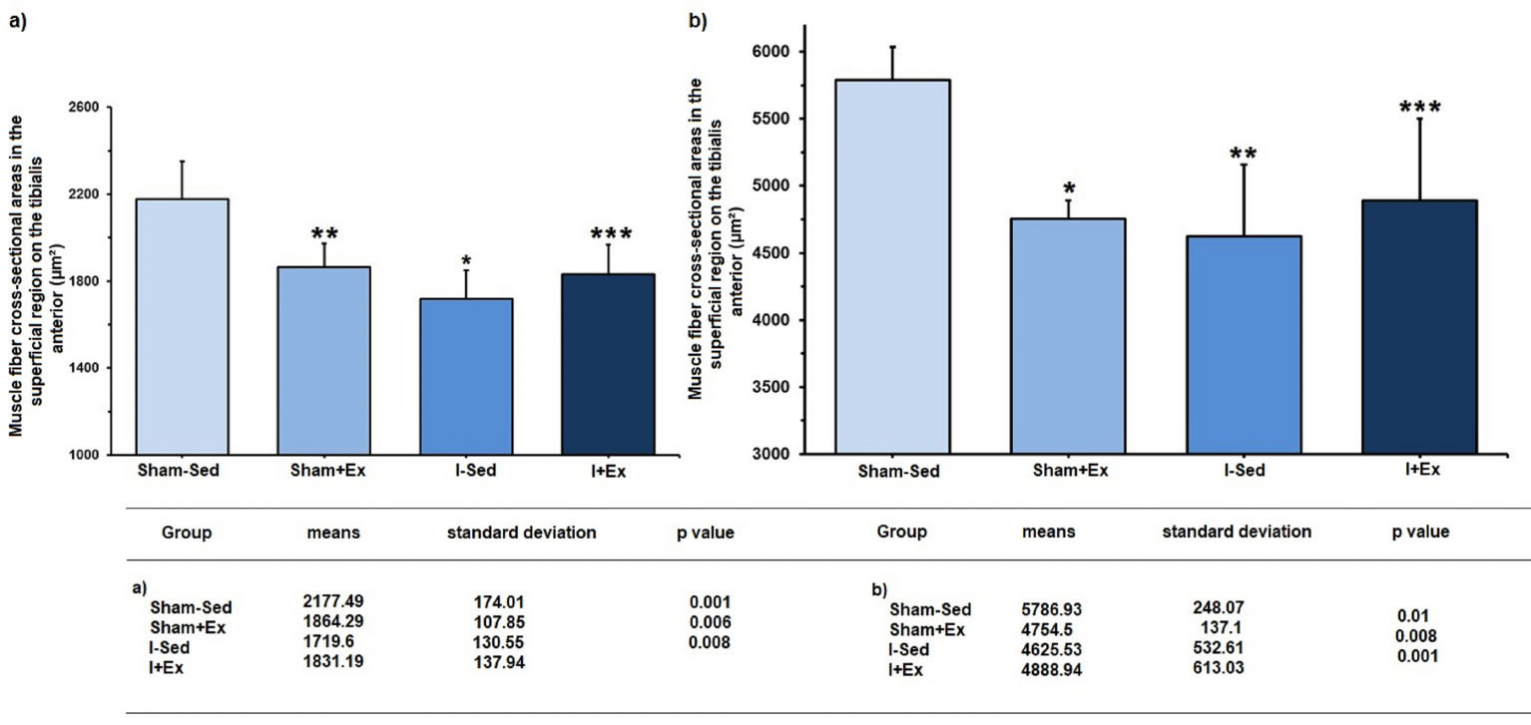
Muscle fiber cross-sectional areas in the deep region of the tibialis anterior muscle. **a**, Type-I fibers: *P=0.001; **P=0.006; ***P=0.008 *vs* Sham-Sed group. **b**, Type-II fibers: *P=0.01; **P=0.008; ***P=0.001 *vs* Sham-Sed group (ANOVA, followed by the Newman-Keuls *post-*test). Sham-Sed: Sedentary control group; Sham+Ex: Control group exercised before the sham surgery; I-Sed: Sedentary ischemic group; I+Ex: Ischemic group exercised before occlusion of the middle cerebral artery surgery.

The data obtained for the area of type-I and type-II fibers in the superficial and deep regions of the tibialis anterior muscle are shown in Figures 3 and 4.

### Volumetric proportion

The histological evaluation of the biceps brachii, soleus, and tibialis anterior muscles of the different groups showed a predominance of muscle fibers with preserved morphology, in a cross-sectional polygonal aspect, individually involved by the endomysium, and organized in fascicles by the perimysium.

Considering the volumetric proportion of the biceps brachii constituents, no significant difference was observed regarding the number of counted points in the cytoplasm of the normal muscle fibers in the different groups (P=0.42). Nonetheless, muscle fiber nuclei density was significantly higher in the exercised ischemic group compared to the other groups (P=0.03). The proportion of interstitium (endomysium, perimysium, and epimysium) did not show significant differences among the different groups (P=0.35).

Regarding the vascular elements, a higher frequency was observed in the vascularization of the biceps brachii in the exercised ischemic group compared to the other groups (P=0.001).

Mast cells, leukocytes, and altered fibers were identified only in the sedentary ischemic group (I-Sed), and nuclei displacement into the muscle fiber was visualized only in the exercised controls (Sham+Ex).

**Figure 5:**
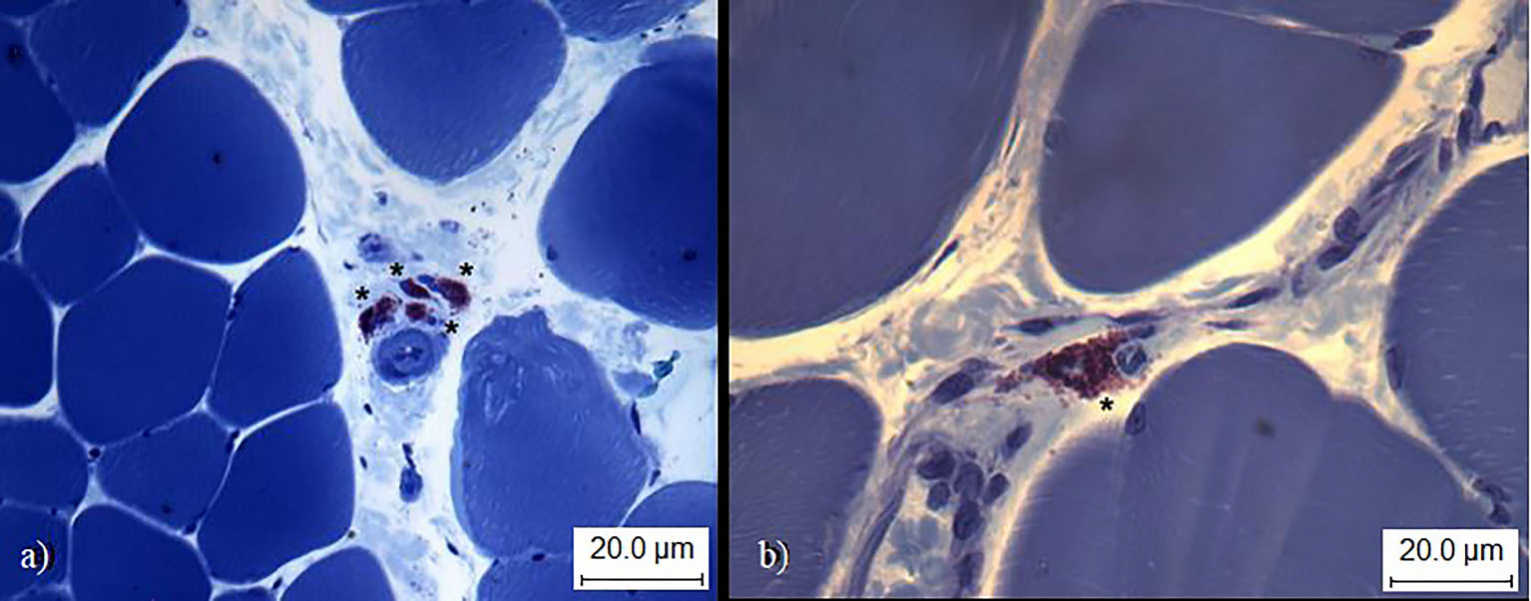
Soleus muscle of sedentary ischemic animals. **a**, mast cells (asterisks) located in the interstitium; **b**, mast cell (asterisk). Staining: toluidine blue+sodium borate. Bar: 20.0 μm.

**Figure 6:**
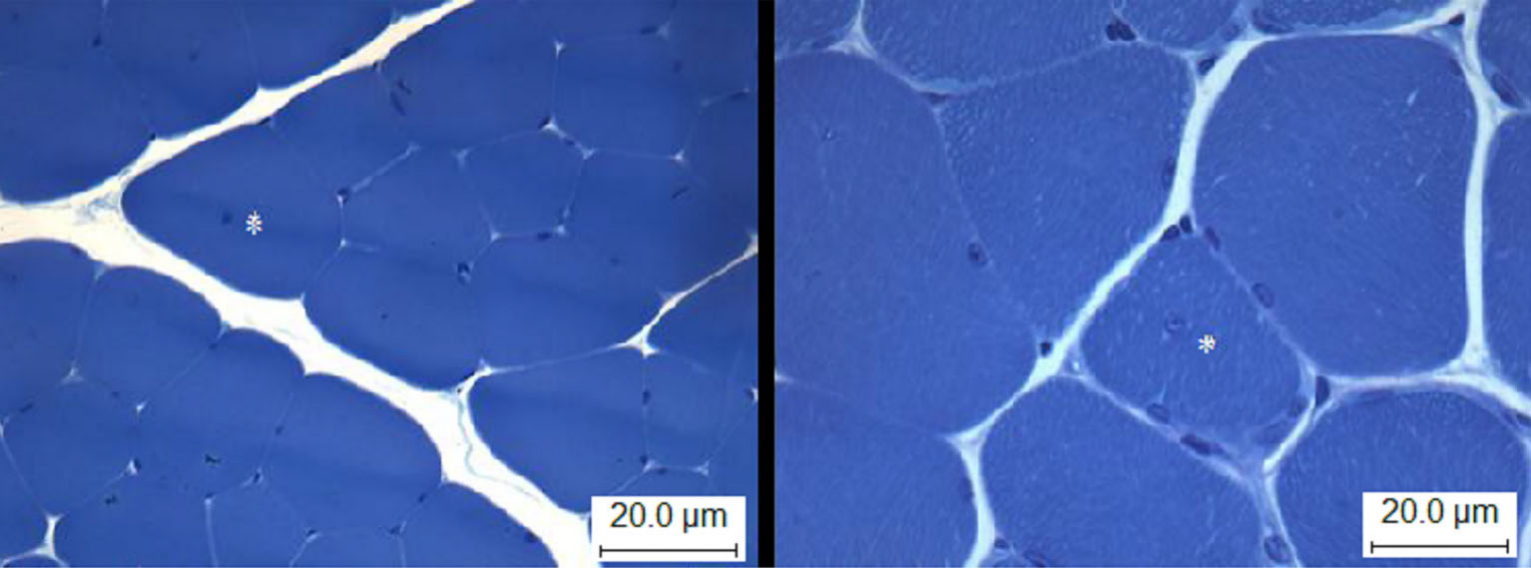
Soleus muscle of sedentary ischemic animals. Muscle fiber cross-sectional area. *Nuclei displacement towards the interior of the muscle fiber. Staining: toluidine blue+sodium borate. Bar: 20.0 μm.

In the soleus muscle, the volumetric ratio did not show a significant difference concerning the number of counted points in the cytoplasm of normal muscle fibers in the different group (P=0.68). Regarding the proportion of fiber nuclei, a higher frequency was observed in the ischemic animals and the exercised control group compared to the sedentary controls (P=0.0002). The interstitial frequency was reduced in the exercised control group compared to the sedentary control group (P=0.04). Considering the vascularization of the soleus muscle, a higher proportion of vascular elements was observed in the ischemic animals compared to the controls (P=0.04). Mast cells were found only in the ischemic groups (I-Sed and I+Ex), as well as altered fibers. Leukocytes and displaced nuclei were observed in the Sham+Ex, I-Sed, and I+Ex groups, as shown in Figures 5 and 6.

In the tibialis anterior muscle, significant changes were not observed in the cytoplasmic ratio of normal fibers (P=0.11), fiber nuclei (P=0.34), and interstitium (P=0.08) among the different groups. Regarding vascularization, a higher frequency of vascular elements was observed in the exercised groups (Sham+Ex: 0.32±0.19 units and I+Ex: 0.32±0.13 units; P<0.05) compared to the sedentary groups (Sham-Sed: 0±0 units and I-Sed: 0.04±0.19 units; P=0.002). Mast cells were observed only in the sedentary ischemic group (I-Sed), and leukocytes and altered fibers were identified only in the ischemic animals (I-Sed and I+Ex), more notably in the I-Sed group. Fiber nuclei displacement was present in groups Sham-Ex, I-Sed, and I-Ex.

## Discussion

A significant decrease in the body weight of animals with cerebral ischemia was observed in the present study. These data corroborate those reported by Modo et al. (22) and Choe et al. (9), where animals with cerebral ischemia presented a lower rate of daily feed intake after OMCA surgery compared to the controls. As a consequence, the authors verified that the ischemic animals, during the first week following OMCA, exhibited significantly lower body weight in comparison with the control group.

The superior performance of the Sham+Ex group is due, in addition to the effects of exercise training, to the motor impairment of animals with cerebral ischemia. When compared to the control animals, the Sham+Ex group exhibited a significant reduction in relation to the Sham-Sed group, suggesting that exercise actually improved the motor performance of these animals. Regarding those that underwent OMCA, the I+Ex group presented a significant reduction in the number of committed errors compared to the sedentary animals of the I-Sed group, showing the neuroprotective effect of exercise training. In the study by Ding et al. (23), the authors reported that animals with cerebral ischemia displayed motor impairment of the anterior and posterior limbs from the 5th to the 28th day after reperfusion, shown in the false step test and the parallel bars test.

The trained animals with cerebral ischemia presented a significant reduction in the number of errors committed during the false step test. These data may be associated with a substantial decrease in the cerebral infarction area, as reported by Damazio et al. (14). According to Marin et al. (24), in addition to reducing the area of cerebral infarction in animals that undergo OMCA, exercise training produces improvement in motor performance and coordination.

The data indicated that exercise training improved the performance of the exercised control animals when crossing the bars and that the OMCA surgery generated motor deficits in the hind legs of the ischemic group. Additionally, the exercise practiced by the I+Ex group did not guarantee better performance compared to the I-Sed group. The pre-surgery training program was not sufficient to ensure the enhanced performance of the trained animals at the end of the experiment since the smaller number of errors observed in these animals (Sham+Ex and I+Ex) in relation to the sedentary animals (Sham-Sed and I-Sed) was not significant.

In spite of the considerable reduction in the area of the soleus muscle fibers and certain regions of the biceps brachii and tibialis anterior muscles of the ischemic animals, the cytoplasmic, normal, and interstitial muscle fibers showed no significant alterations compared to the animals of the control groups.

Muscular atrophy is observed in experiments involving the OMCA model, in which a reduction in the cross-sectional area of muscle fibers is present. Voos and Ribeiro (25) reported that cerebral ischemia affects the entire flexor musculature of the hemiparetic thoracic limb, causing atrophy by decreasing the use of these muscles. In the present study, motor impairment was observed in the front right paws of the ischemic animals, shown by their inferior performance in the false step test at the end of the experiment.

Regarding the density of muscle fiber nuclei, an increase was observed in the ischemic animals compared to the controls, mainly in the soleus muscle but also in the biceps brachii, and more prominently in the exercised ischemic group (I+Ex). Vascular elements were more frequent in the ischemic animals than in the controls. Among the ischemic groups, those who underwent treadmill training (I+Ex) presented a higher proportion of vascular elements than the sedentary (I-Sed) animals.

The data obtained when measuring the areas of the biceps muscle type-I and type-II fibers demonstrated hypertrophy of both fiber types in the superficial region of the muscle in the ischemic animals (I-Sed and I+Ex), whereas atrophy was detected throughout the soleus muscle. Scherbakov and Doehner (26) reported that skeletal muscles have a high adaptive potential, although the mechanisms of atrophy and phenotypic change after cerebral ischemia are not fully understood. They also reported that patients who have sustained cerebral ischemia may present increases in the MHC type-II isoform and decreases in the MHC type-I isoform. Nevertheless, these results are quite controversial, since several studies have not shown a significant difference in the proportion between type-I and type-II fibers (27). Discrepancies in fiber frequency were not observed in the present research in the superficial region of the biceps brachii regarding the control and ischemic groups. However, there was a difference between trained and sedentary animals. In the deep region of the biceps brachii, a higher frequency of type-II fibers was observed only in the exercised ischemic group (I+Ex).

Scelsi et al. (28) reported a reduction in the diameter of type-I and type-II fibers of the tibialis anterior muscle in the paretic limbs of individuals with cerebral ischemia. Chang et al. (29), on the other hand, described a significant decrease in the mass of the tibialis anterior muscle in ischemic rats compared with the controls. Herein, our data indicated selective atrophy of type-I fibers in the sedentary ischemic group (I-Sed) in the superficial region of the muscle. Exercise training prior to OMCA surgery led to an increase in fiber area and aided in preventing muscular atrophy in the animals of the I+Ex group. In the deep region, however, a reduction in the area of type-I and type-II fibers was observed in the ischemic groups compared to the sedentary controls. Therefore, the present data, in addition to those obtained in previous studies, indicated atrophy of type-I (superficial region) and type-I and type-II (deep region) fibers of the tibialis anterior muscle of ischemic rats.

Yan et al. (30), Simoneau et al. (31), and Green et al. (32) reported that exercise training may cause an increase in the proportion of type-I fibers in skeletal muscles, but only in cases where exercise is practiced during long periods, as in the case of athletes who undergo intense training for years. In the present experiment, an exercise protocol of moderate intensity was used for a relatively long period of time. The exercise training may have caused an increase in the proportion of type-I fibers in the exercised groups.

Muscle fiber hypertrophy in cerebral ischemia models has not been well elucidated, although some studies have detected such alteration in some muscles (16). The exercise performed before cerebral ischemia (I+Ex group) was efficient in preventing muscular atrophy of type-I fibers in the deep biceps brachii region, given that these animals presented a larger fiber area compared to the sedentary ischemic group (I-Sed).

Mast cells and leukocytes were visualized mainly in the sedentary and trained ischemic animals. A more significant number of immune cells was expected in the muscle interstitium of the ischemic animals. However, these data can be explained by the low frequency of altered fibers and the relatively long duration of the experiment. Nuclei displacement towards the fiber center, evident primarily in the soleus and tibialis anterior muscles, can also be indicative of muscle regeneration. These centrally positioned nuclei were present only in the groups where altered fibers and/or mast cells and leukocytes were observed.

Vascular elements were more frequent in the ischemic groups than the controls. Among the ischemic animals submitted to treadmill training (I+Ex), a higher proportion was observed compared with the sedentary (I-Sed) group. In the study by Yan et al. (30), angiogenesis was observed in the brain and skeletal muscles of trained rats.

Therefore, the main alterations that occurred in the biceps brachii, soleus, and tibialis anterior muscles were possibly due to the normal processes of muscle regeneration caused by the detriment in neuromuscular control. Significant effects of exercise were observed for the proportion of vascular elements, suggesting that angiogenesis may have occurred in the evaluated skeletal muscles.

However, the present study has limitations because it did not perform analysis of VEGF expression or investigation of functional capillary density to better understand the angiogenesis process.

In conclusion, our results suggest that the angiogenesis process may have influenced muscle recovery and reduced muscle atrophy of type-I fibers in the animals that exercised prior to cerebral ischemia, in addition to improving the coordination and motor performance of these animals.
